# A novel variant on chromosome 6p21.1 is associated with the risk of developing colorectal cancer: a two-stage case-control study in Han Chinese

**DOI:** 10.1186/s12885-016-2843-7

**Published:** 2016-10-18

**Authors:** Chunxiao Xu, Dan Zhou, Feixia Pan, Yi Liu, Dandan zhang, Aifen Lin, Xiaoping Miao, Yaqin Ni, Duo Lv, Shuai Zhang, Xiaobo Li, Yimin Zhu, Maode Lai

**Affiliations:** 1 Department of Epidemiology and Biostatistics, School of Public Health, Zhejiang University, 866 Yuhangtang Road, Hangzhou, 310058 Zhejiang Province China; 2 Department of Chronic Non-Communicable Diseases Control and Prevention, Zhejiang Provincial Center for Disease Control and Prevention, Hangzhou, Zhejiang Province China; 3 Department of Pathology, School of Medicine, Zhejiang University, 866 Yuhangtang Road, Hangzhou, 310058 Zhejiang China; 4 Key Laboratory of Disease Proteomics of Zhejiang Province and Department of Pathology, School of Medicine, Zhejiang University, Hangzhou, China; 5 Human Tissue Bank, Taizhou Hospital of Zhejiang Province, Zhejiang, China; 6 Medical Research Center, Taizhou Hospital of Zhejiang Province, Zhejiang, China; 7 Department of Epidemiology and Biostatistics, School of Public Health, Tongji Medical College, Huazhong University of Science and Technology, Hubei, China; 8 Department of Computer Science and Technology, College of Engineering, Lishui University, Lishui, Zhejiang China

**Keywords:** Colorectal cancer, Genetic variant, Inflammation, HSP90

## Abstract

**Background:**

Genes in inflammatory pathways play a pivotal role in the development of colorectal cancer. We conducted a two-stage case-control study and aimed at screening the colorectal cancer-associated genetic variations in inflammatory genes.

**Methods:**

Twenty-three candidate variants were genotyped in 952 primary colorectal cancer cases and 875 cancer-free controls from eastern China. Promising single nucleotide polymorphisms were further genotyped in 518 cases and 554 controls from middle China. Expression quantitative trait loci and differential gene expression analyses were performed for the associated gene.

**Results:**

rs2282151 presented consistently significant associations with the risk of colorectal cancer in both stages (odds ratio (95 % confidence interval) = 1.30 (1.16–1.46), risk allele = C, *P*_combined_ = 8.9E-6). Gene expression quantitative trait loci analyzes uncovered consistent *cis*-regulatory signals which showed that the C allele of rs2282151 was associated with increased expression level of heat shock protein 90 alpha family class B member 1 (*HSP90AB1*). Then we found that the mRNA expression levels of *HSP90AB1* were significantly higher in tumor tissues than normal tissues (fold-change = 1.83) in 28 pairs of colorectal tissue samples. The expression difference was consistent with data from online datasets. Additionally, we observed notable peaks of H3K27ac and H3K4me3 near the first intron of *HSP90AB1* using ChIP-seq data from multiple cell lines (including HCT116).

**Conclusions:**

Our findings indicate that the C allele of the novel colorectal cancer-associated variant rs2282151 is associated with increased expression levels of *HSP90AB1*, which is expressed higher in colorectal tumor tissues than in normal tissues.

**Electronic supplementary material:**

The online version of this article (doi:10.1186/s12885-016-2843-7) contains supplementary material, which is available to authorized users.

## Background

Colorectal cancer (CRC) is the third most common malignant neoplasm and the fourth leading cause of cancer deaths in the world, with an estimated 1,360,000 diagnoses and 694,000 deaths from this disease worldwide in 2012 [1]. In China, the incidence of CRC ranked fourth in all cancer sites with the crude rate of 23.0/100,000 in 2010, and the mortality rate for CRC was ranked fifth in cancer-related deaths in all cancer sites with the rate of 11.1/100,000 [2].

Previous studies have shown that inflammatory processes played an important role in the etiology of cancers including CRC [3–6]. It is acknowledged that patients with inflammatory bowel disease, including ulcerative colitis and Crohn’s disease, are at increased risk of developing CRC [7, 8]. Evidence from previous findings has also indicated that nonsteroidal anti-inflammatory drugs such as aspirin could reduce the risk of CRC [9, 10] and adenomatous polyps [11]. Genetic factors contribute to the development of CRC through an inflammatory process. To date, several genes in the inflammatory pathway have been identified to be linked to colorectal cancer, including *NFKBIA*, *NFKBIB*, *NFKBIE*, *IL6*, *STAT3*, *CXCL12*, *COX2*, and *PPARG* etc. [12–16]. Normally, the major transcription regulator NFκB is inactivated in the cytoplasm by binding to its inhibitor IκB. Phosphorylation and degradation of IκB proteins lead to the release and nuclear localization of NFκB proteins, which then initiate transcription of those target genes of NFκB [17]. Dysfunction of IκB can lead to the inhibition or constitutive activation of NFκB [18]. IκBs mainly consist of IκBα, IκBβ, and IκBγ, coded by *NFKBIA*, *NFKBIB*, and *NFKBIE*, respectively. Interleukin-6 (IL-6), an important pro-inflammatory cytokine, is found to be associated with many types of cancers. Associations between the *IL6* gene polymorphisms and CRC have been observed by previous studies [19–22]. STAT3, a nuclear transcription factor downstream of the IL-6 family, has also been associated with CRC [23]. The persistent activation of STAT3 mediates tumour-promoting inflammation [16]. The expression of activated STAT3 was related to tumor invasion and poor prognosis of CRC [24]. The chemokine CXCL12 has been found to play a decisive role in tumor progression [25], and it directs leukocyte migration through interactions with its receptor CXCR4 [26, 27]. CXCL12/CXCR4 interactions might constitute a promising target for specific treatment interventions [28]. In this study, we mainly focused on the polymorphism of the candidate genes including *NFKBIA*, *NFKBIB*, *NFKBIE*, *IL6*, *STAT3*, and *CXCL12*.

Variations in these genes may affect chronic inflammation levels, which have been linked to the risk of cancers including CRC. Recently, genome-wide association studies (GWAS), as a powerful strategy, has been focusing on common variants, and uncovered several point mutations associated with CRC in different populations including Asian [29–32]. However, few replicable variants located in inflammation-related genes have been reported in these studies. The small effect of the variants on the inflammation genes and the strict false-positive control in GWAS could be the reasonable explanations. Many true-positive variants probably are neglected in GWAS with *P*-values (in discovery stage) ranging from 0.05 to 1.0E-4, which could hardly be selected into replication stages. Evidences from a study by Zhang and colleagues successfully replicated six novel CRC-associated loci which showed *P*-values ranging only from 0.002 to 0.03 in the discovery stage [32]. Candidate gene strategy with a reasonable hypothesis to narrow down the candidate single-nucleotide polymorphisms (SNPs) could be reconsidered after replications for the top signals in GWAS.

We conducted a two-stage case-control study between CRC risk and genetic variants of inflammation-related genes in Han Chinese. The promising tagSNPs from the discovery stage were genotyped for further replication in an independent population. Fine-mapping and functional annotation were performed for replicated tagSNP using online data sources. Differential gene expression analysis was conducted in 28 CRC patients with tissue samples and validated using online datasets.

## Methods

### Study subjects

In this study, two independent case-control studies were performed in Han Chinese. The discovery phase consisted of 952 newly diagnosed cases with primary CRC and 875 cancer-free controls. Cases were consecutively and systematically recruited with same diagnostic criteria by trained investigators between 2006 and 2011 at The First Affiliated Hospital, Sir Run Run Shaw Hospital of Medical College, Zhejiang University, and Zhejiang Taizhou Hospital (Eastern China). The pathologic diagnoses of CRC were evaluated by pathologists from biopsy reports. Patients with familial adenomatous polyposis and hereditary non-polyposis CRC were excluded. The healthy controls were recruited from a population-based cross-sectional study via a large-scale physical examination in the medical center of the Third People’s Hospital of Xiaoshan Zhejiang (Eastern China), from July 2010 to July 2011. Physical examination and face-to-face survey were conducted by trained investigators. Subjects were excluded from the control group if they had 1) cancers, 2) a family history of cancers or polyposis, or 3) serious chronic liver, lung, heart, or kidney disorders were also excluded.

In the replication stage, considering the regional representation, 517 CRC cases were recruited from Taihe Hospital in Shiyan, Hubei Province (Middle China), between 2011 and 2012. 550 controls in the second stage were randomly selected from a community-based cancer screening program for early detection of cancer conducted in the same regions during the same time. The diagnostic criteria and the exclusion criteria were the same as mentioned above. Written informed consent was obtained from all subjects, and the study protocol was approved by the Institutional Review Board of Zhejiang University, School of Medicine.

### tagSNP selection, genotyping, and imputations

Genomic DNA was extracted from peripheral blood cells using a TACO Nucleic Acid Automatic Extraction System (GeneReach Biotechnology Corp., Taiwan). All of the TagSNPs were genotyped via the Illumina Human-OmniExpress 760 k chip (Illumina, San Diego, CA, USA) in the discovery stage, as described previously [33]. SNPs with a minor allele frequency (MAF) > 0.05 in the HapMap Han Chinese in Beijing (CHB) population were included in the study. Only one SNP was chosen if two or more SNPs were observed to be in high linkage disequilibrium (*r* ^2^ ≥ 0.80) in the study. A total of 23 tagSNPs near the six genes passed the quality control procedures of the GWAS chips (including call rate > 95 %, *P*_Hardy-Weinberg Equilibrium_ > 0.05, etc.) and were chosen in the first stage of the study. In the replication phase, two promising SNPs were genotyped by the TaqMan allelic discrimination assay using a LightCycler® 480 Instrument (Roche, Mannheim, Germany). All the primer and probe sequences are available upon request. In replication stage, 10 % double-blind duplicates and negative controls were included in each 384-well plate.

Post-quality-control GWAS data were used for imputation. We imputed ungenotyped SNPs via IMPUTE2 (http://mathgen.stats.ox.ac.uk/impute/impute_v2.html) with the worldwide haplotype reference data of 1092 individuals from the 1000 Genomes Project Phase I integrated variant set (v3, released in March 2012). SNPs with info score quality estimates < 0.8 were excluded from the analyses. Finally, 4,642,479 SNPs were used for fine-mapping. The genotyping call rates for these polymorphisms were all above 95 %.

### Online data collection

*Cis*-expression Quantitative Trait Loci (eQTL) analyses, i.e., the associations between genotype and mRNA expression in *cis*-regulation, were acquired from the MuTHER project in multiple tissues [34] and a meta-analysis in non-transformed peripheral blood samples from seven studies (EGCUT, InCHIANTI, Rotterdam Study, Fehrmann, HVH, SHIP-TREND, and DILGOM) [35]. Evidence of differential gene expression (tumor tissue vs normal tissue) was collected from multiple sources including 32 pairs of samples using the Illumina Hiseq 2000 RNA-seq platform from TCGA, 97 pairs of samples using Affymetrix Human Genome U219 Array from Adria Closa and colleagues [36] (GSE44076), and 103 pairs of samples using the Illumina Hiseq 2000 RNA-seq platform from the SYSCOL human CRC project [37]. The genetic architectures surrounding replicated SNPs were assessed using the Encyclopedia of DNA Elements (ENCODE) database from the The University of California, Santa Cruz (UCSC) genome browser [38]. Annotated peaks of histone modification (H3K27ac, H3K4me3) and POLR2A were called from multiple cell lines including the CRC cell line HCT116. Using data from the NIH Roadmap Epigenomics Mapping Consortium, four other colon and rectal tissue samples (including one colonic mucosa, one sigmoid colon, and two rectal mucosa samples) were used for histone modification annotations as supplements.

### Quantitative Polymerase Chain Reaction (qPCR) analyzes

Twenty-eight pairs of tissue samples (tumor tissue and paired adjacent normal tissue) were collected from CRC patients during surgery at Zhejiang Taizhou Hospital (April 2010 to April 2011). mRNA samples were extracted from these fresh tissue samples and reverse-transcripted into cDNA for storage. qPCR was performed to determine the expression levels of the target genes using the ABI 7900HT real time PCR system (Applied Biosystems, Foster City, CA). *GAPDH* was used as house-keeping gene in the current study.

### Statistical analysis

The Hardy-Weinberg Equilibrium test for each SNP was assessed by goodness-of-fit *χ* ^*2*^ test in the control groups. The *χ* ^*2*^ test was used to evaluate the significant of differences in the distribution of gender. The *t*-test was used to examine the differences between cases and controls in age. In the current analyses, the minor allele of each SNP was considered as the effect allele and the major allele was considered as the reference allele. Logistic regression models were used to explore the associations between genotypes and risk of CRC in the additive model (i.e., the genotypes were assigned “0”, “1”, “2”, according to the number of effect alleles), and the odds ratio (OR) and its 95 % confidence interval (95 % CI) were estimated. SNPs with significant associations with CRC in the discovery stage after false discovery rate control by the Benjamini–Hochberg (BH) procedure were genotyped in the replication stage. In order to control the potential confounding effect by age, we additionally performed analyses in subgroups stratified by age (categorized as age < 50, between 50 and 70, and age > 70 years). Meta-analyses were carried out to combine the results from different stages. A fixed-effect model was used when there was no indication of heterogeneity (Cochran’s Q-statistic *P* > 0.05). Wilcoxon’s signed ranks test was carried out for the comparison of mRNA expression levels between the tumor and paired normal tissues. Statistical analyses were performed using the SAS for Windows software (version 9.2, SAS Institute Inc., Cary, NC). The region around the replicated tagSNP was plotted using LocusZoom based on the ASN population in hg19 coordinates [39]. Histone modification annotations were visualized using the UCSC genome browser.

## Results

### Basic characteristics of study subjects

Totally, 1469 cases and 1425 cancer-free controls were recruited in this study. The characteristics of the study subjects in the two stages are summarized in Table 1. In both stages, no significant difference was found between cases and controls in gender. As the cases were significantly older compared with controls, we additionally performed age-stratification analyses.

**Table 1:** Basic characteristics of the samples in the two-stage case-control study

	Discovery stage		Replication stage		Combined	
	Cases (n, %)	Controls (n, %)	Cases (n, %)	Controls (n, %)	Cases (n, %)	Controls (n, %)
Gender						
Female	432 (45.4)	418 (47.8)	232 (44.9)	224 (40.7)	664 (45.2)	642 (45.1)
Male	520 (54.6)	457 (52.2)	285 (55.1)	326 (59.3)	805 (54.8)	783 (54.9)
Age						
Mean ± SD (years)	62.8 ± 12.3	55.3 ± 11.8	55.1 ± 10.7	45.2 ± 11.7	60.1 ± 12.3	51.4 ± 12.7
Tumor location						
Colon	453 (47.9)		232 (44.9)		685 (46.6)	
Rectum	471 (49.5)		223 (43.1)		694 (47.2)	
Missing	28 (2.6)		62 (12.0)		90 (6.1)	

*Abbreviation*: *n* number of samples

### Associations of candidate tagSNPs with CRC

All the 23 candidate SNPs in this study population were consistent with HWE (*P* > 0.05) in the controls in both stages. The associations between candidate tagSNPs with CRC risk is presented in Additional file 1: Table S1. Four SNPs, including *STAT3* rs1053005, *CXCL12* rs1029153, *NFKBIA* rs1022714, and *NFKBIE* rs2282151 were found nominally associated (*P* < 0.05) with CRC risk via logistic regression in an additive model adjusted for age and gender (Additional file 1: Table S1). Two SNPs, i.e., rs1022714 (*P* = 0.003) and rs2282151 (*P* = 0.002), were significantly associated with CRC after false discovery rate correction (Table 2).

**Table 2:** Associations of SNPs with CRC risk in the discovery stage, replication stage and combined samples

Gene	SNP	Chr	Position (hg19)	Genotype	Effect allele	Discovery stage			Replication stage			Combined		
						Case/control	OR (95 % CI)^a^	*P* ^a^	Case/control	OR (95 % CI)^a^	*P* ^a^	Case/control	OR (95 % CI)^a^	*P* ^a^
*NFKBIA*	rs1022714	14	35871407	GG	A	550/450	0.78 (0.67–0.92)	0.003 0.045^*^	291/293	1.12 (0.90–1.41)	0.306			
				GA		359/362			191/200					
				AA		43/63			27/37					
*NFKBIE*	rs2282151	6	44226195	TT	C	329/352	1.24 (1.08–1.42)	0.003 0.034^*^	136/202	1.39 (1.14–1.70)	0.001	465/554	1.30 (1.16–1.46)	8.9E-6
				TC		456/401			281/251			737/652		
				CC		159/118			91/72			250/190		

*Abbreviation*: *Chr* chromosome, *OR* odds ratio, *95 % CI* 95%confidence interval

^a^Logistic regression were performed in additive model, adjusted for age and sex

^*^ *P*-value after FDR control

After replications, rs2282151 in *NFKBIE* was found to be significantly associated with CRC risk in the same direction as in the discovery stage (Table 2). The C allele of rs2282151 in *NFKBIE* was associated with a significantly increased risk of CRC (OR = 1.24; 95 % CI: 1.08–1.42 in the discovery stage (*P* = 0.002) and 1.39 (95 % CI: 1.14–1.70) in replication stage (*P* = 0.001), respectively. Combined statistical analysis showed that rs2282151 had a per-allele OR of 1.30 (95 % CI: 1.16–1.46) with the combined *P*-value of 8.9E-6. No significant heterogeneity was observed between the discovery and replication stages (*P*_heterogeneity_ = 0.72). Besides the additive model, we evaluated the association of rs2282151 and CRC in dominant and recessive models. According to the results shown in Additional file 2: Table S2, the combined effect sizes were closed among the additive (OR = 1.30 (1.16–1.46)), the dominant (OR = 1.41 (1.19–1.66), and the recessive model (OR = 1.37 (1.10–1.71)). No statistical association was observed for rs1022714 and CRC in the replication stage (*P* = 0.306).

### Age-stratified analyses of rs2282151 and CRC

Because of the higher mean of ages in the case group, we classified the samples into three groups (age < 50, between 50 and 70, and age > 70 years) and performed age-stratified logistic regressions (controlling for gender and age as covariates). Results showed that the effect was consistent with the age < 50 and 50–70 years group, while no difference was found in the > 70 years group in discovery stage (Table 3). In combined analyses (combined with discovery and replication stages), the OR and 95 % CI were 1.33 (1.09, 1.62), 1.33 (1.14, 1.55), and 1.17 (0.81, 1.68) in the < 50, 50–70 and ≥ 70 age groups, respectively. The combined *P*-value was 6.2E-6 (combined with three age groups).

**Table 3:** Age-stratified analyses of rs2282151 with CRC risk in the discovery, replication stage and combined samples

		Discovery stage			Replication stage			Combined	
	Case/control	OR (95 % CI)	*P* ^a^	Case/control	OR (95 % CI)	*P* ^a^	Case/control	OR (95 % CI)	*P* ^a^
Age < 50	158/246	1.39 (1.03, 1.88)	0.029	151/390	1.28 (0.97, 1.68)	0.080	309/636	1.33 (1.09, 1.62)	0.006
50 ≤ Age < 70	520/552	1.24 (1.04, 1.48)	0.015	342/143	1.71 (1.22, 2.39)	0.002	862/695	1.33 (1.14, 1.55)	5.2E-4
Age ≥ 70	274/77	1.00 (0.68, 1.47)	0.992	24/16	4.48 (1.43, 14.02)	0.010	298/93	1.17 (0.81, 1.68)	0.405
Combined	952/875	1.24 (1.07, 1.42)	0.003	517/549	1.49 (1.21, 1.84)	1.8E-4	1469/1424	1.31 (1.17, 1.47)	6.2E-6

*Abbreviation*: *OR* odds ratio, *95 % CI* 95 % confidence interval

^a^Logistic regression were performed in additive model, adjusted for age and sex

### Fine-mapping for rs2282151 and function annotations

**Fig. 1:**
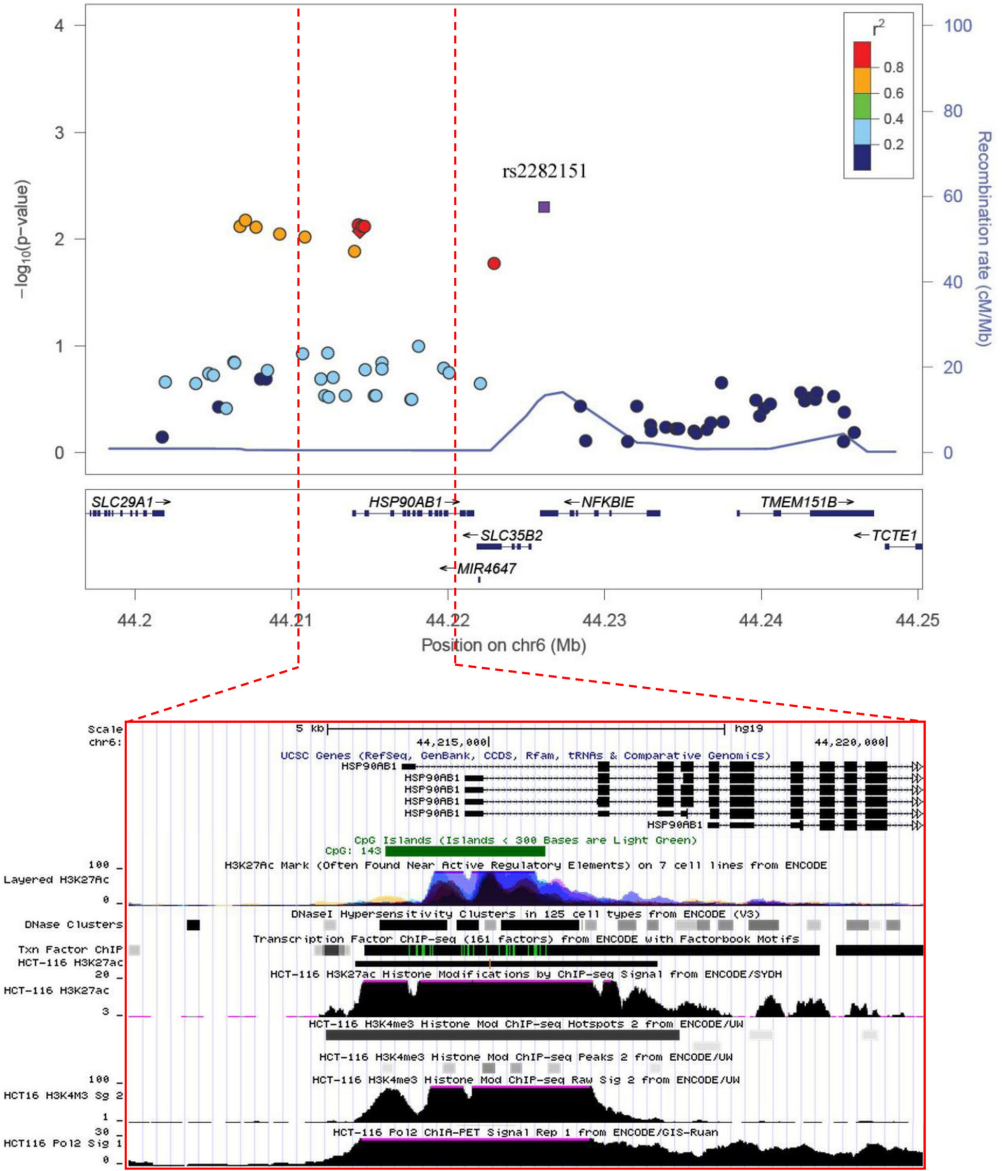
Regional plot of the variant rs2282151 with the *P*-value from discovery stage in hg19 coordinate plotted by the online tool locusZoom. Annotations on *HSP90AB1* were identified using The University of California, Santa Cruz (UCSC) genome browser. The variant with the highest -log10 *P* value is colored *purple* and identified by its rs number. All other loci are represented reflecting the level of correlation with the variant. The linkage disequilibrium is estimated using Asian samples from the 1000 Genome Project. The name and location of genes in the UCSC genome browser are shown. A 10 kb window near the 5′ end of *HSP90AB1* was annotated with CpG island, histone modification (H3K27ac, H3K4me3) and POLR2A peaks from multiple cell lines including HCT116 from the Encyclopedia of DNA Elements project

**Fig. 2:**
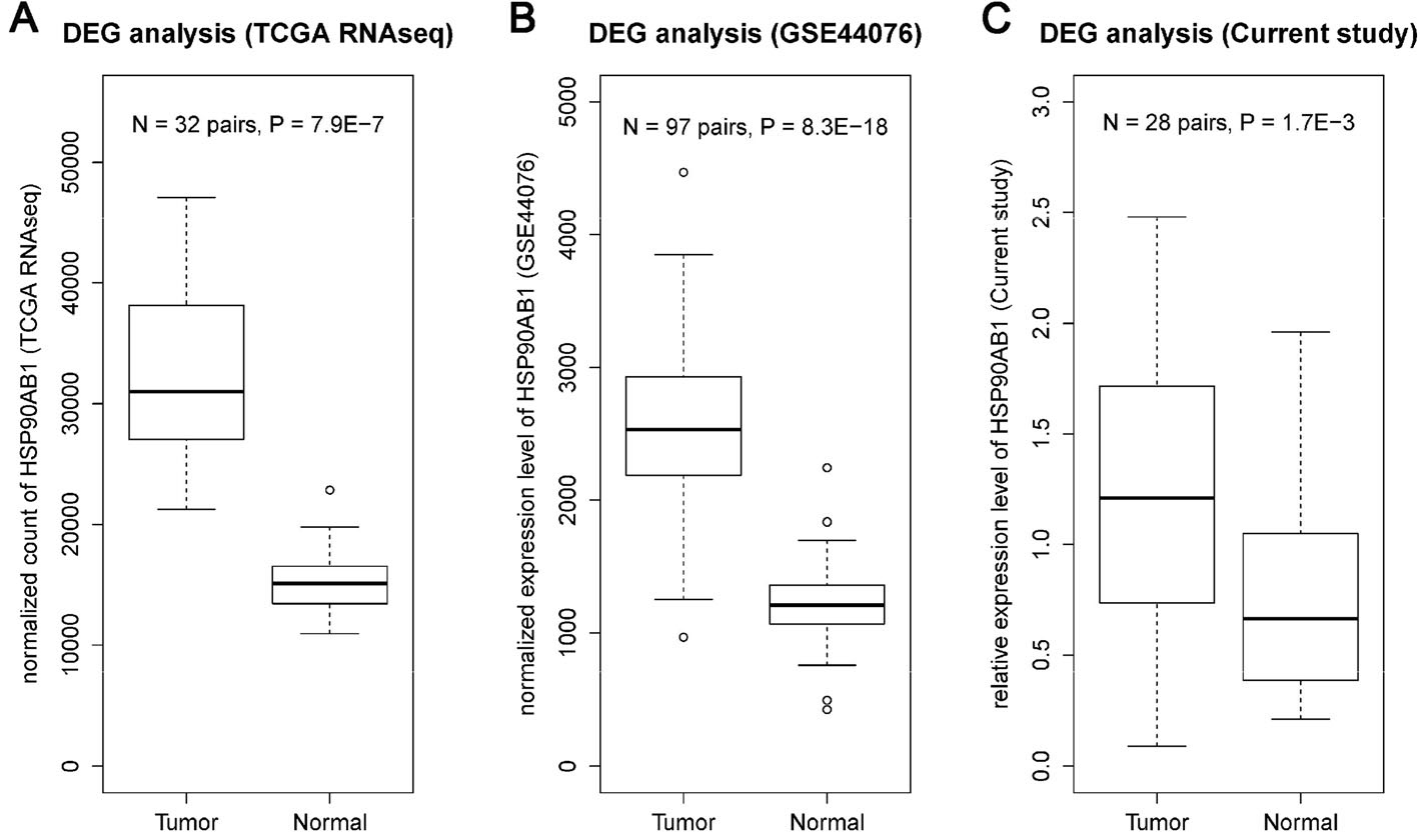
The differential gene expression (DGE) analyses revealing that the mRNA expression level of *HSP90AB1* in colorectal tumor tissue was significantly higher than paired normal tissue samples. We obtained the expression levels of colorectal tumor and paired normal tissue samples from **a** The Cancer Genome Atlas (TCGA) project using RNAseq platform (32 pairs), **b** GSE44076 database using Affymetrix Human Genome U219 Array (97 pairs), and **c** Current study performed via quantitative Polymerase Chain Reaction using *GAPDH* as housekeeping gene (28 pairs). *P*-values were calculated using two related sample Wilcoxon signed rank test. Abbreviations: *N* = Number of pairs

**Fig. 3:**
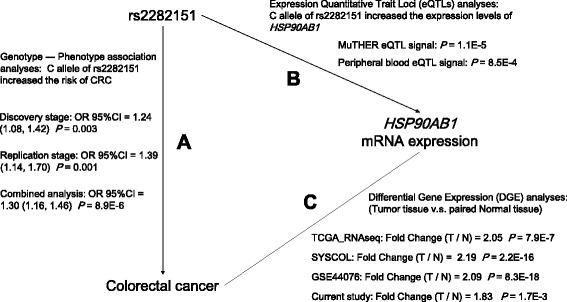
Three-way associations among the variant, mRNA expression and the risk of developing colorectal cancer. Evidence for **a** genotype and phenotype associations were concluded from the two-stage case-control study. **b** Expression quantitative trait loci (eQTL) analyses showed the association between genotypes and mRNA expression levels. **c** Differential gene expression (DGE) analyses revealed the upregulated expression levels in colorectal tumor tissues than paired normal tissues. Abbreviations: OR = odds ratio; CI = confidence interval; MuTHER = Multiple Tissue Human Expression Resource; TCGA = The Cancer Genome Atlas; SYSCOL = Systems Biology of Colorectal Cancer; T / *N* = Tumor / Normal

The replicated signal of rs2282151 at 6p21.1 is located in the 3′UTR of *NFKBIE*. As the regional plot shows (Fig. 1), in this region, 12 SNPs in high linkage disequilibrium (*r* ^2^ > 0.5) with rs2282151 also displayed promising evidence of association (*P* = 0.008–0.027 via imputed genotypes) in the discovery stage. Three protein-coding genes (*HSP90AB1*, *SLC35B2*, and *NFKBIE*) and a non-coding RNA (*MIR4647*) were contained in this region. eQTL analysis was conducted using the online databases. Evidence suggested that the C allele in rs2282151 was significantly associated with increased mRNA expression level of *HSP90AB1* in skin tissue (MuTHER). The effect size (beta and its 95 % CI) of the *cis*-regulatory signal was 0.09 (0.50, 0.13), *P* = 1.1E-5. Consistently, the C allele of rs2282151 was associated with increased level of *HSP90AB1* in a combined analysis using expression levels from whole blood cells (*P* = 8.5E-4). Then we compared the difference of mRNA expression levels of *HSP90AB1* in colorectal tumor and paired normal samples using online data sources. Tumor tissues had significantly higher expression levels than paired normal tissues using databases from TCGA (32 pairs of samples), SYSCOL (103 pairs of samples), and GSE44076 (97 pairs of samples). As shown in Figs. 2 and 3, the *HSP90AB1* was significantly highly expressed in tumor tissues than normal tissues. The expression fold changes (tumor/normal) were ranged from 2.05 to 2.19. We further performed differential gene expression analysis in 28 paired samples from Zhejiang Taizhou hospital and found consistent results (expression fold changes (tumor/normal) was 1.83, *P* = 1.7E-3). However, the associations between the genotype of rs2282151 and the expression of *HSP90AB1* did not reach significance in colon tissues (*P* > 0.05). No consistent signals were found between rs2282151 and other genes in *cis*-regulatory.

Using the UCSC genome browser, we observed that five of the SNPs tagged by rs2282151 (linkage disequilibrium *r* ^2^ > 0.5) were located in the first intron of *HSP90AB1*. As shown in Fig. 1, these five SNPs are also located in a CpG island in this region (USCS annotation). We noticed that of the five SNPs, the genotypes of rs10948128 (flanking sequence 5′ GCG[C/G]GTC 3′) and rs3757283 (flanking sequence 5′ GGA[A/C]GAA 3′) influence the existence of CpG sites which probably leads to different methylation levels on these CpG sites. Additionally, we observed remarkable histone modification peaks (H3K27ac and H3K4me3) in eight different cell lines including HCT116 via ChIP-seq data from the ENCODE project (Fig. 1). We observed that the histone modification signals (H3K27ac, H3K4me3) were consistent with the signals in the HCT-116 cell line (Additional file 3: Figure S1). Besides these SNPs, five other SNPs tagged by rs2282151 were found to be located upstream of *HSP90AB1*, while no additional evidence was found.

## Discussion

We identified the tagSNP rs2282151 on chromosome 6p21.1 as a susceptibility marker for CRC by a two-stage case-control study. This tagSNP is located in an linkage disequilibrium block covering two inflammation-related genes (*NFKBIE* and *HSP90AB1*). The C allele of rs2282151 was associated with increased mRNA expression level of *HSP90AB1*, which expressed higher in CRC tumor tissues than paired normal tissues.

In this two-stage study, 1469 cases and 1425 controls were recruited. All of the study subjects are Han Chinese (an ethnic group native to East Asia), which constitute approximately 92 % of the population of Mainland China and about 18 % of the entire global human population. We investigated 23 tagSNPs in six genes in the inflammation pathway for their associations with CRC.

The additive model was used for screening CRC-associated SNPs. Results showed that two tagSNPs were significantly associated with CRC in discovery stage after false discovery rate control. After the replication stage, rs2282151 maintained a consistently significant association with increased risk of CRC. Under different inheritance models, the allelic dose-response trends of rs2282151 indicated that the additive model was suitable for the novel variant. The tagSNP rs2282151 located in an linkage disequilibrium block in 6p21.1. Four genes including two inflammation-related genes *NFKBIE* and *HSP90AB1* were located in this linkage disequilibrium block. In order to find clues for the causal gene, we obtained and analyzed the results of eQTL analyses using online data from MuTHER project. The project developed detailed genomic and transcriptome data from three disease-relevant tissues (adipose, lymphoblastoid cell lines and skin) originating from a cohort of 856 phenotyped twins from the TwinsUK adult registry [34]. Results suggested C allele of rs2282151 was significantly associated with increased expression levels of *HSP90AB1* in skin tissues. This eQTL signal was replicated in whole blood samples with a consistent direction of effect [35]. However, probably because of the limited sample size (*N* = 28), the association was not replicated in colon tissue samples. Beside the eQTL signals, we found significantly higher expression levels of *HSP90AB1* in CRC tumor than paired normal samples in all three online databases. The expression difference was replicated in 28 pairs of tissue samples via qPCR in Han Chinese.

Then we annotated the position of SNPs tagged by rs2282151 and found five SNPs located in the first intron of *HSP90AB1*. Using UCSC genome browser, we found remarkable peaks of H3K27Ac and H3K4me3, which indicated the activity of the transcription. All these five candidate causal SNPs were located in the peak of H3K27Ac. Notably, the polymorphisms of rs10948128 and rs3757283 (two of the five SNPs) were involved with the existence of CpG sites. These CpG-site-related SNPs are called “cgSNP” or “meSNP” and probably influence the methylation levels on the CpG site (i.e., probably methylation QTL, mQTL) [40–42]. Both of the two SNP-CpG pairs are located in a CpG island overlapped with the first intron of *HSP90AB1*.

Integrated with the evidence shown above, we drew a three-way association triangle among the genotype of the tagSNP rs2282151, the expression levels of *HSP90AB1*, and the risk of CRC. As shown in Fig. 3, the C allele of rs2282151 was associated with increased risk of CRC. Meanwhile, the C allele of rs2282151 was associated with increased expression level of *HSP90AB1*, which was expressed higher in CRC tumor tissue than normal tissue.

The heat shock protein 90 kDa alpha (cytosolic), class B member 1 (*HSP90AB1*), encodes a member of the heat shock protein 90 (HSP90) family, which is involved in signal transduction, protein folding and degradation, and morphological evolution [43–45]. *HSP90AB1* is necessary for the shuttle of client proteins between the cytoplasm and nucleus [46, 47]. Client proteins including IκB kinase (IKK) complex are required for HSP90 as a regulator of NFκB signaling by its general involvement in IKK activation and by its role in IKK homeostasis [48]. A study by Nagaraju showed that the inhibition of HSP90 downregulates both HIF-1a and NFκB leading to the inhibition of epithelial to mesenchymal transition (EMT), motility, and invasiveness in CRC [49]. Other studies also have shown that *HSP90AB1* was upregulated in lung cancer and melanoma tumor [50–52]. The up-regulation in lung cancer was associated with poor survival [51]. Since HSPs (including HSP90) are frequently upregulated in tumor tissues and tumor growth is supported by HSP proteins [43], inhibition of HSPs is a promising strategy for therapy. Inhibition of *HSP90AB1* function is a potential target for CRC treatment according to current studies. However, functional studies are needed to demonstrate the role of *HSP90AB1* in carcinogenesis.

However, our current study has some limitations. The first one is the different distribution of age between the cases and controls. In order to control the potential confounding effect by age, we conducted age-stratified association analyses. The results showed the associations between tagSNP and CRC in the < 70 years’ groups, while no significant association was observed in the ≥ 70 years’ groups. One possible reason was the limited sample size in this subgroup. Another explanation was the increased attributions of environmental factors and aging in carcinogenesis compared with genetic factors in older populations. Lack of data such as smoking, drinking behaviors and family history of related diseases was another limitation of the current study.

## Conclusion

In conclusion, this study identifies a novel variant of rs2282151, which is associated with the risk of developing CRC in Han Chinese. This association between rs2282151 and CRC is probably mediated by the expression of the inflammatory-related gene *HSP90AB1*, which is upregulated in colorectal tumor tissues. Further studies are warranted to explore the potential mechanism.

## Additional files

Additional file 1: Table S1. The basic information of the 23 candidate tagSNPs and the results of the association study in discovery stage of these tagSNPs. (DOCX 17 kb)
Additional file 2: Table S2. Associations of rs2282151 with CRC risk in the discovery stage, replication stage and combined samples in different genetic models. (DOCX 15 kb)
Additional file 3: Figure S1. The histone modification signals for *HSP90AB1*. The 5′ end of *HSP90AB1* was annotated using histone modification signals of H3K27ac, H3K4me1, H3K4me3, and H3K9ac in A (colonic mucosa), B (sigmoid colon), C (rectal mucosa 1), and D (rectal mucosa 2). (TIFF 1992 kb)

## Abbreviations

CI: Confidence interval
CRC: Colorectal cancer
eQTL: Expression quantitative trait loci
GWAS: Genome-wide association studies
HSP90AB1: Heat shock protein 90 kDa alpha, class B member 1
MAF: Minor allele frequency
NFκB: Nuclear factor kappa-light-chain-enhancer of activated B cells
OR: Odds ratio
SNP: Single nucleotide polymorphism
