# Supplementary material for: A novel variant on chromosome 6p21.1 is associated with the risk of developing colorectal cancer: a two-stage case-control study in Han Chinese
Source: BMC Cancer. 2016 Oct 18;16:807. doi: 10.1186/s12885-016-2843-7 (PMC5069896; doi:10.1186/s12885-016-2843-7)
Supplement: Additional file 1: Table S1. — The basic information of the 23 candidate tagSNPs and the results of the association study in discovery stage of these tagSNPs. (DOCX 17 kb) [file 12885_2016_2843_MOESM1_ESM.docx]

**Supplementary table 1. The basic information of the 23 candidate tagSNPs and the results of the association study in discovery stage of these tagSNPs.**

| Gene | tagSNPs | Chr | Position(hg19) | Effect allele | N | OR^†^ | *P*^†^ |
| --- | --- | --- | --- | --- | --- | --- | --- |
| *NFKBIE* | **rs2282151** | **6** | **44226195** | **C** | **1815** | 1.24 (1.1,1.38) | **0.002** |
|  | rs3799962 | 6 | 44228452 | G | 1813 | 1.08 (0.94,1.22) | 0.291 |
|  | rs2233434 | 6 | 44232920 | G | 1823 | 1.06 (0.86,1.26) | 0.574 |
| *IL6* | rs2069837 | 7 | 22768027 | G | 1821 | 1.05 (0.87,1.23) | 0.579 |
|  | rs2066992 | 7 | 22768249 | C | 1827 | 1.00 (0.84,1.16) | 0.990 |
| *CXCL12* | rs266093 | 10 | 44866208 | C | 1820 | 0.90 (0.72,1.08) | 0.231 |
|  | rs2839696 | 10 | 44866628 | A | 1826 | 0.76 (0.49,1.03) | 0.062 |
|  | **rs1029153** | **10** | **44867146** | **G** | **1823** | 0.81 (0.65,0.97) | **0.010** |
|  | rs3740085 | 10 | 44867769 | C | 1824 | 0.86 (0.66,1.06) | 0.144 |
|  | rs1801157 | 10 | 44868257 | A | 1788 | 1.07 (0.91,1.23) | 0.419 |
|  | rs266089 | 10 | 44869427 | A | 1801 | 1.08 (0.88,1.28) | 0.421 |
|  | rs266087 | 10 | 44871062 | G | 1827 | 0.95 (0.81,1.09) | 0.450 |
|  | rs17881575 | 10 | 44871346 | A | 1827 | 0.94 (0.72,1.16) | 0.532 |
|  | rs7075976 | 10 | 49614277 | G | 1824 | 1.01 (0.87,1.15) | 0.925 |
| *NFKBIA* | rs8904 | 14 | 35871217 | A | 1826 | 1.13 (0.99,1.27) | 0.084 |
|  | **rs1022714** | **14** | **35871407** | **A** | **1827** | 0.78 (0.62,0.94) | **0.003** |
|  | rs1957106 | 14 | 35873770 | A | 1822 | 0.95 (0.79,1.11) | 0.518 |
| *STAT3* | **rs1053005** | **17** | **40465910** | **G** | **1823** | 1.22 (1.06,1.38) | **0.008** |
|  | rs3816769 | 17 | 40498273 | G | 1826 | 1.18 (0.98,1.38) | 0.085 |
| *NFKBIB* | rs11879872 | 19 | 39395331 | G | 1820 | 1.02 (0.88,1.16) | 0.833 |
|  | rs3136642 | 19 | 39398416 | A | 1819 | 0.92 (0.76,1.08) | 0.248 |
|  | rs3136644 | 19 | 39398631 | G | 1818 | 0.97 (0.83,1.11) | 0.632 |
|  | rs3136645 | 19 | 39398649 | G | 1820 | 0.97 (0.83,1.11) | 0.672 |

Abbreviation: Chr=chromosome, N=number of samples successfully genotyped, OR=odds ratio, SE=standard error.

^†^ Logistic regression were performed in additive model, adjusted for age and sex.
