# Supplementary material for: A novel variant on chromosome 6p21.1 is associated with the risk of developing colorectal cancer: a two-stage case-control study in Han Chinese
Source: BMC Cancer. 2016 Oct 18;16:807. doi: 10.1186/s12885-016-2843-7 (PMC5069896; doi:10.1186/s12885-016-2843-7)
Supplement: Additional file 2: Table S2. — Associations of rs2282151 with CRC risk in the discovery stage, replication stage and combined samples in different genetic models. (DOCX 15 kb) [file 12885_2016_2843_MOESM2_ESM.docx]

**Supplementary Table 2. Associations of rs2282151 with CRC risk in the discovery stage, replication stage and combined samples in different genetic models.**

| Stages |  | Additive model | | |  | Dominant model | |  | Recessive model | |
| --- | --- | --- | --- | --- | --- | --- | --- | --- | --- | --- |
|  |  | TT (0) | TC (1) | CC (2) |  | TT (0) | TC/CC (1) |  | TT/TC (0) | CC (1) |
| Discovery stage | N of case | 329 | 456 | 159 |  | 329 | 615 |  | 785 | 159 |
|  | N of control | 352 | 401 | 118 |  | 352 | 519 |  | 753 | 118 |
|  | case % | 48.3% | 53.2% | 57.4% |  | 48.3% | 54.2% |  | 51.0% | 57.4% |
|  | OR (95%CI) | 1.24 (1.08-1.42) | | |  | 1.33 (1.09-1.63) | |  | 1.34 (1.02-1.76) | |
|  | *P* | 3.0E-03 | | |  | 4.9E-03 | |  | 3.3E-02 | |
|  |  |  |  |  |  |  |  |  |  |  |
| Replication stage | N of case | 136 | 281 | 91 |  | 136 | 372 |  | 417 | 91 |
|  | N of control | 202 | 251 | 72 |  | 202 | 323 |  | 453 | 72 |
|  | case % | 40.2% | 52.8% | 55.8% |  | 40.2% | 53.5% |  | 47.9% | 55.8% |
|  | OR (95%CI) | 1.39 (1.14-1.70) | | |  | 1.58 (1.19-2.12) | |  | 1.44 (0.99-2.09) | |
|  | *P* | 1.0E-03 | | |  | 1.8E-03 | |  | 5.9E-02 | |
|  |  |  |  |  |  |  |  |  |  |  |
| Combined | N of case | 465 | 737 | 250 |  | 465 | 987 |  | 1202 | 250 |
|  | N of control | 554 | 652 | 190 |  | 554 | 842 |  | 1206 | 190 |
|  | case % | 45.6% | 53.1% | 56.8% |  | 45.6% | 54.0% |  | 49.9% | 56.8% |
|  | OR (95%CI) | 1.30 (1.16-1.46) | | |  | 1.41 (1.19-1.66) | |  | 1.37 (1.10-1.71) | |
|  | *P* | 8.9E-06 | | |  | 4.4E-05 | |  | 5.0E-03 | |
