# Supplementary figures and images for: A novel variant on chromosome 6p21.1 is associated with the risk of developing colorectal cancer: a two-stage case-control study in Han Chinese
Source: BMC Cancer. 2016 Oct 18;16:807. doi: 10.1186/s12885-016-2843-7 (PMC5069896; doi:10.1186/s12885-016-2843-7)

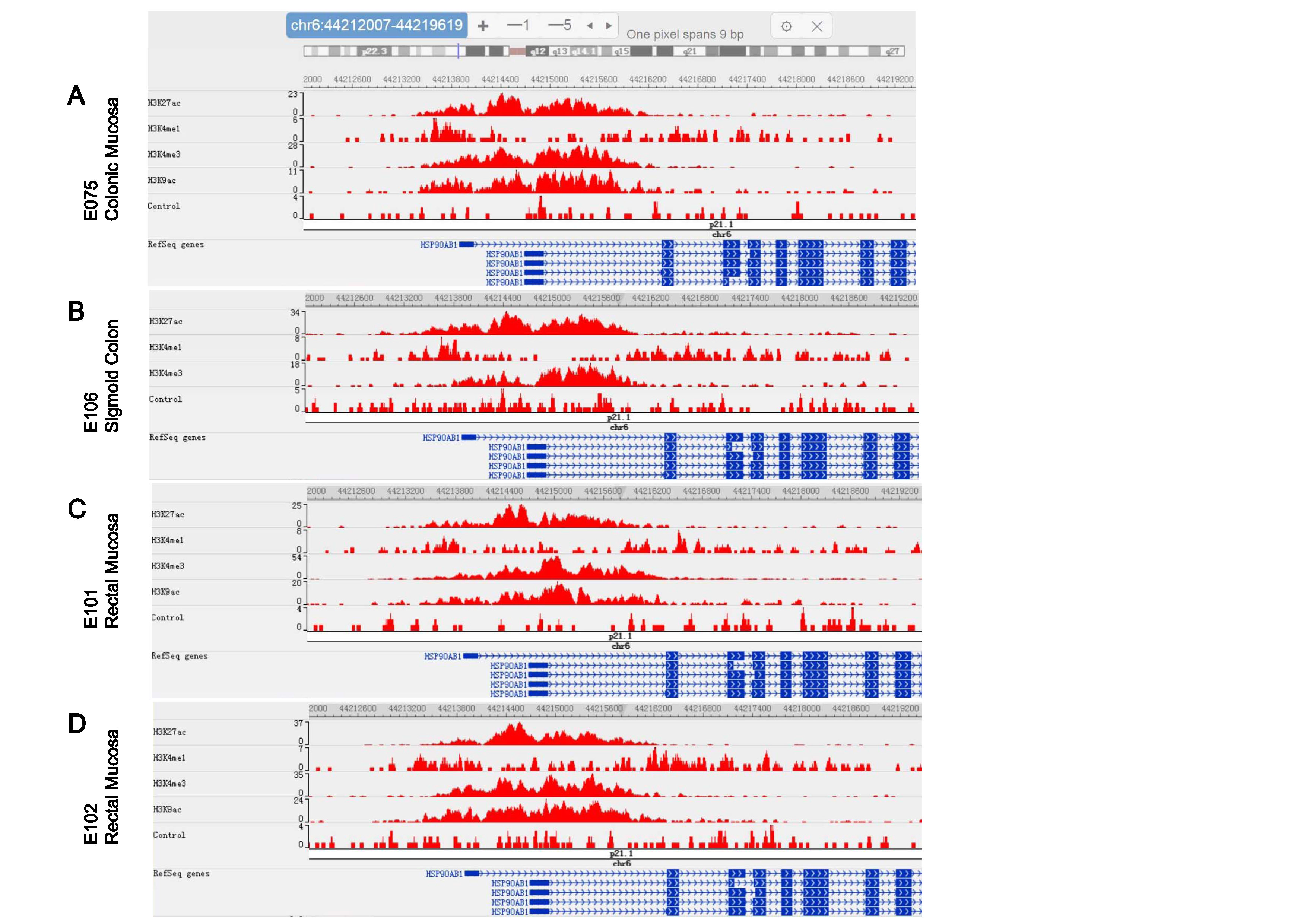

Supplement: Additional file 3: Figure S1. — The histone modification signals for HSP90AB1. The 5′ end of HSP90AB1 was annotated using histone modification signals of H3K27ac, H3K4me1, H3K4me3, and H3K9ac in A (colonic mucosa), B (sigmoid colon), C (rectal mucosa 1), and D (rectal mucosa 2). (TIFF 1992 kb) [file 12885_2016_2843_MOESM3_ESM.tiff]
